# A Rare Case of Diffuse Extra‐Cranial Schwannomatosis of the Trunk and Limbs

**DOI:** 10.1002/ccr3.72554

**Published:** 2026-04-10

**Authors:** Bernadette Pedun, Ivaan Pitua, Felix Bongomin, Samuel Bugeza, Alex Bakenga, Valeria Nabbosa

**Affiliations:** ^1^ Uganda Cancer Institute Kampala Uganda; ^2^ Department of Radiology and Radiotherapy, School of Medicine, College of Health Sciences Makerere University Kampala Uganda; ^3^ School of Medicine, College of Health Sciences Makerere University Kampala Uganda; ^4^ Department of Internal Medicine Gulu Regional Referral Hospital Gulu Uganda; ^5^ Department of Medical Microbiology and Immunology Gulu University Gulu Uganda

**Keywords:** case report, CT scan, neurofibromatosis 1 and 2 (NF1, NF2), peripheral nerve sheath tumor (PNST), schwannoma

## Abstract

Schwannomas are benign peripheral nerve sheath tumors (PNSTs) arising from myelin sheaths, with schwannomatosis characterized by multiple lesions without neurofibromatosis type 1 (NF1) or type 2 (NF2) stigmata. Malignant transformation is rare but documented. We report a 44‐year‐old patient with multiple painful, slow‐growing recurrent masses in the right axilla, bilateral inguinal regions, and right lumbar area; these masses were initially noted early in 2017. Surgery was done with subsequent recurrence 6 years after initial diagnosis and surgery. Contrast‐enhanced CT scans done in 2023 revealed heterogeneously enhancing, well‐defined ovoid masses, some with spinal connections, consistent with schwannomas. Multiple biopsies of different sites at different time periods were consistent with histology that confirmed the diagnosis of schwannomatosis, with no NF1 or NF2 features. Histopathology results are attached. The patient underwent palliative 1st line chemotherapy with noted regression of some of the masses, though surgical resection remains the standard treatment. The last contact with the patient prior to writing this case was at the time of chemotherapy. This case highlights schwannomatosis as a rare, painful condition distinct from NF1 and NF2. CT imaging is vital in low‐resource settings for diagnosis, emphasizing the need for careful differentiation from other PNSTs due to distinct management and prognosis.

## Key Clinical Message

Schwannomatosis is a rare condition that requires clear differentiation from NF1 and NF2, as accurate diagnosis directly guides appropriate management. In resource‐limited settings, CT imaging in combination with histopathology plays a central role in identifying characteristic features, making precise recognition essential for avoiding misclassification and ensuring correct treatment planning.

## 1 Introduction

Schwannomas are common benign peripheral nerve sheath tumors (PNSTs) originating from myelin sheaths, classified by the World Health Organization as PNSTs [1, 2]. These slow‐growing, encapsulated lesions range from 2 to 15 cm and are eccentric to nerve fascicles, with rare malignant transformation or primary malignant forms reported [3, 4]. Schwannomas occur sporadically or in association with neurofibromatosis type 1 (NF1) or type 2 (NF2) due to genetic mutations [5, 6]. They typically present in the third to fifth decades, with equal gender distribution and an annual incidence of 0.4 per 100,000 [4, 7]. Schwannomas are often asymptomatic until they compress nerves, causing paraesthesia, anesthesia, or pain [8]. They predominantly affect the upper limbs, followed by the trunk and lower limbs [1, 4].

Schwannomatosis is a rare, non‐heritable clinical syndrome characterized by multiple schwannomas without NF2‐associated eighth cranial nerve involvement or NF1 stigmata [9, 10, 11]. Imaging, including ultrasound for superficial lesions and CT or MRI for deeper lesions, is critical for diagnosis, with CT showing heterogeneously enhancing, encapsulated ovoid masses [8]. This report describes the clinical and radiological features of schwannomatosis, focusing on CT findings in a low‐resource setting.

## 2 Case History/Examination

A 44‐year‐old patient presented with several months of painful, gradually enlarging masses in the right axilla, bilateral inguinal regions, right gluteal, and right lumbar areas, accompanied by back pain radiating to the right flank and occasional right‐hand tingling. No motor dysfunction, skin lesions, tinnitus, hearing loss, visual disturbances, or gait issues were reported. Over‐the‐counter medications provided minimal relief. The patient had these similar symptoms about 6 years prior. The patient denied a family history of similar conditions, skin lesions, or bilateral deafness.

**FIGURE 1:**
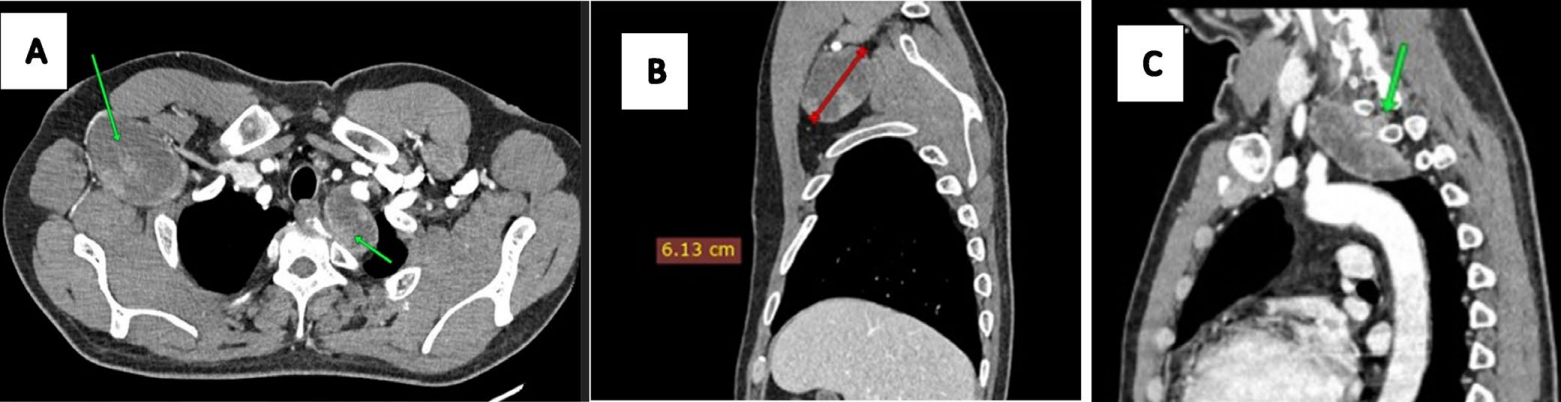
Contrasted CT scans of an axial slice (A), and sagittal reformats (B, C) showing well‐demarcated expansile heterogeneously enhancing soft tissue‐density masses in the right axilla and supero‐posterior mediastinum (green arrows) with mass effect to the surrounding structures, measuring (6.8 × 6.1 × 4.2) cm; the mediastinal mass has a spinal connection as seen by the arrow in C, a sagittal image giving it a characteristic ‘dumb‐bell’ shape.

**FIGURE 2:**
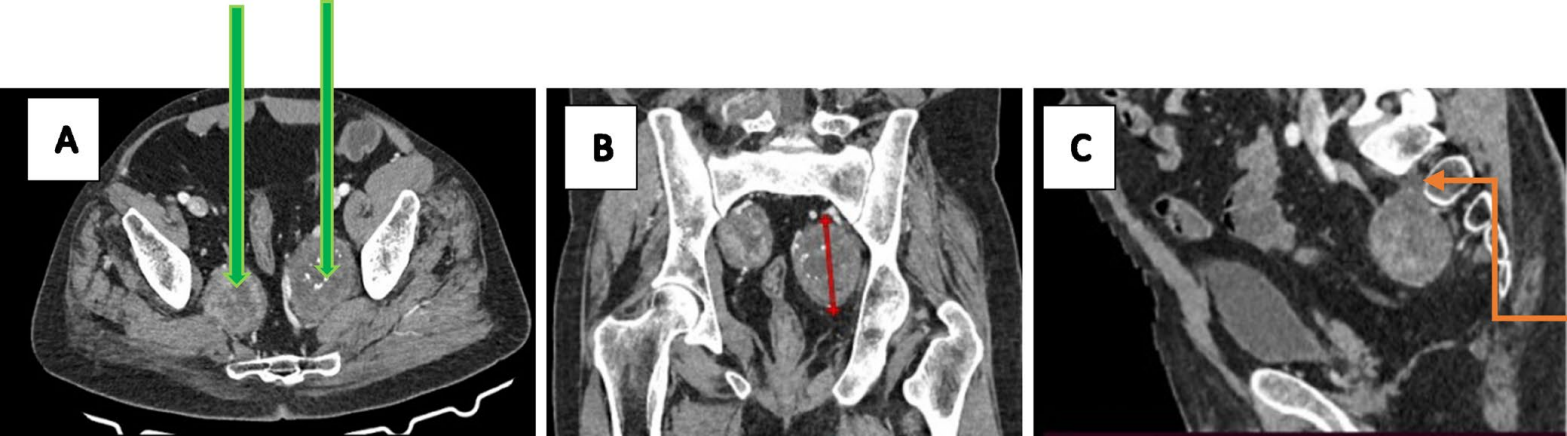
Contrasted CT scan of the pelvis, axial slice (A), coronal (B), and sagittal reformats (C) showing two well‐demarcated heterogeneously enhancing soft tissue‐density masses within the pelvis bilaterally, pre‐sacral in location with a connection to the anterior sacral neuroforamen (orange step arrow in sagittal view).

**FIGURE 3:**
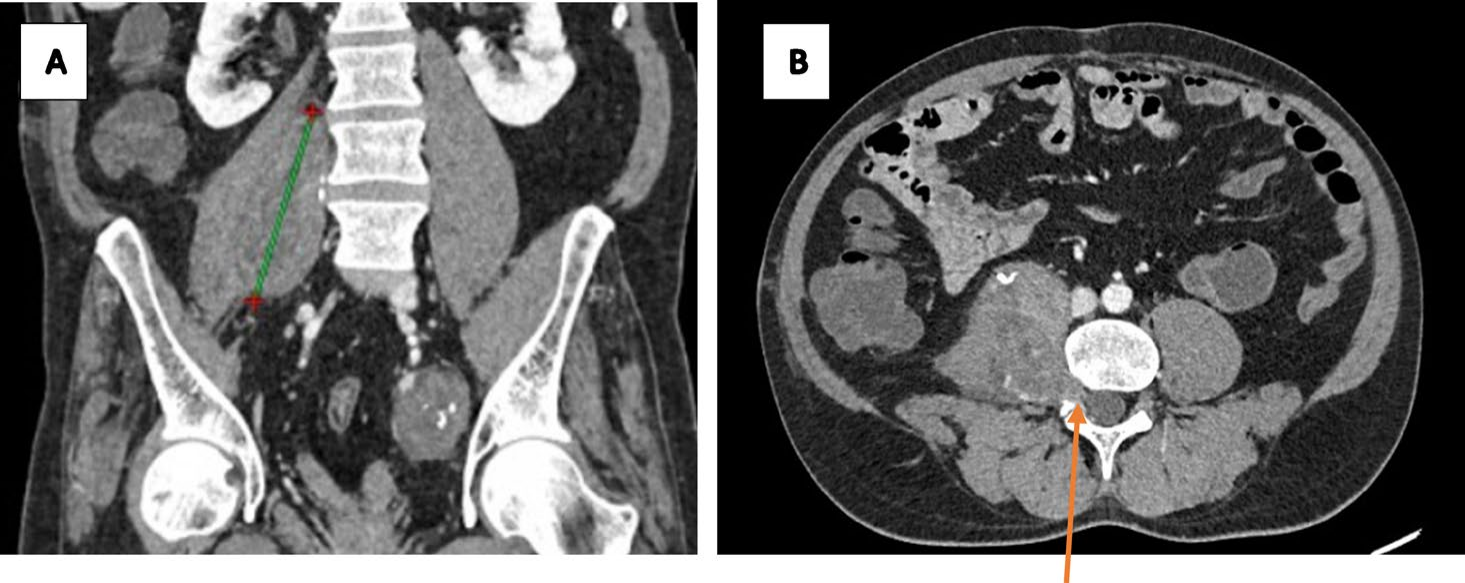
Contrasted CT scan of the abdomen, coronal reconstruction (A) and axial slice (B) showing an ovoid well‐demarcated heterogeneously enhancing mass in the right retro‐peritoneal region, laterally displacing the right psoas muscle, which appears atrophic as compared to the contralateral side. This mass has a connection to a lumbar neuroforamen showing the ‘dumb‐bell’ appearance (orange arrow in the axial image).

**FIGURE 4:**
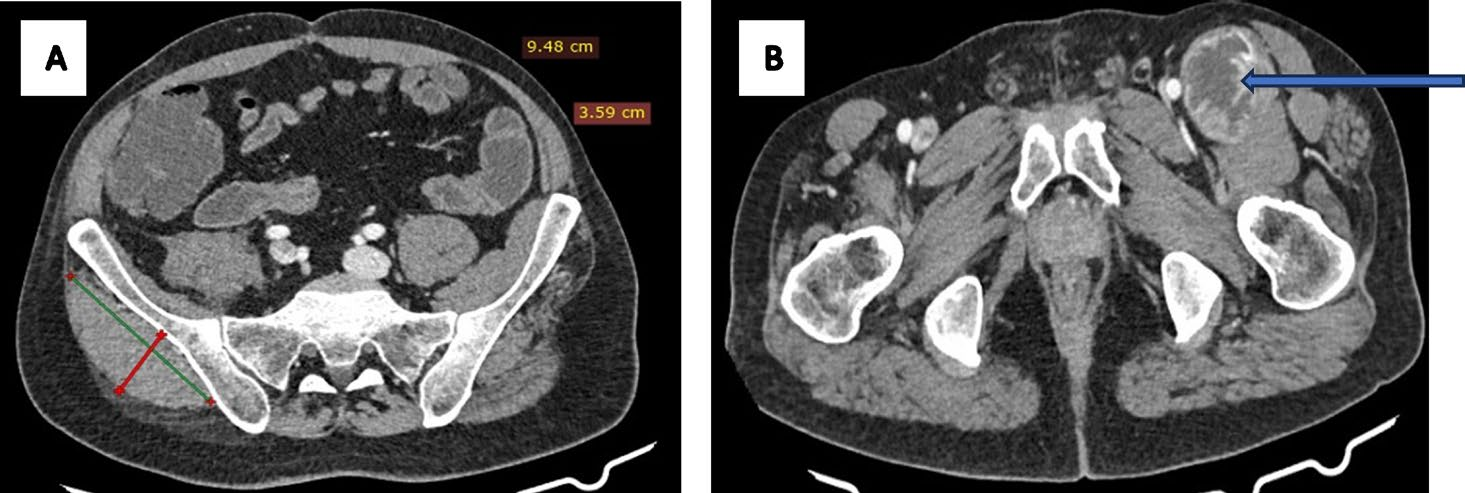
A and B are contrasted CT scans of the pelvis, axial slices showing ovoid and round heterogeneously enhancing soft tissue density masses in the right gluteal (A) and left inguinal area (blue arrow in B).

Physical examination revealed an ambulatory patient with an abducted right arm and a mildly tender, non‐mobile mass in the right axilla. Larger masses were noted in both inguinal regions, right gluteal, and right lumbar areas. Cranial nerve function and motor function were intact, with no skin tags or discoloration. Contrast‐enhanced CT scans of the chest, abdomen, and pelvis demonstrated multiple well‐demarcated, heterogeneously enhancing soft tissue‐density masses. These included a mass in the right axilla and supero‐posterior mediastinum (6.8 × 6.1 × 4.2 cm) with a spinal connection, exhibiting a characteristic “dumb‐bell” shape (Figure 1); bilateral pre‐sacral pelvic masses with anterior sacral neuroforamen connections (Figure 2); an ovoid mass in the right retroperitoneal region displacing the hypotrophic psoas muscle, with a lumbar neuroforamen connection (Figure 3); and masses in the right gluteal and left inguinal areas (Figure 4). Biopsies from the left inguinal, right gluteal, and right retroperitoneal masses confirmed schwannomas, supporting a diagnosis of schwannomatosis. No brain CT scan or MRI was done to radiologically rule out vestibular Schwannomas, even though history and clinical examination were not suggestive of auditory deficits.

## 3 Differential Diagnosis

The patient's presentation of multiple painful masses raised considerations of other PNSTs, including neurofibromas associated with NF1 or NF2‐related schwannomatosis. Malignant peripheral nerve sheath tumors (MPNSTs) were considered due to the possibility of malignant transformation, though less likely given the well‐defined, encapsulated nature of the masses. Soft tissue sarcomas or metastatic lesions were also considered but ruled out by imaging and histopathology. The absence of NF1 stigmata (e.g., café‐au‐lait spots, neurofibromas) and NF2 features (e.g., bilateral vestibular schwannomas) supported schwannomatosis as the primary diagnosis, confirmed by biopsy findings. Prior histopathologies had been done in the past, all in support of schwannomatosis. NF1 was excluded due to painful recurrent nature of the lesions at different concurrent site and no skin pigmentation changes, but we lacked genetic testing such as *SMARCB1* or *LZTR1* mutations to exclude NF2 scientifically. However, the history of symptoms, clinical exam and audiometry did not reveal any auditory deficits, thus pointing towards schwannomatosis, rather that NF variants.

## 4 Conclusion and Results

This case of a 44‐year‐old patient with schwannomatosis highlights the rare presentation of multiple painful Schwannomas without NF1 or NF2 features. CT imaging confirmed heterogeneously enhancing, well‐defined masses in the trunk and limbs, with histopathology verifying the diagnosis. The patient received palliative chemotherapy though surgical resection had been done before with recurrence 6 years later. The chemotherapy regimen consisted of doxorubicin, ifosphamide, mesna and steroids every twenty‐one days, with notable clinical regression of palpable masses noted by the sixth cycle and was yet to do end of treatment imaging. Financial constraints limited access to MRI, the preferred imaging modality, and genetic testing to exclude NF1/NF2 definitively. This case touches the importance of distinguishing schwannomatosis from NF1 and NF2 for appropriate management and the critical role of CT in resource‐limited settings. Ongoing multidisciplinary care aims to optimize outcomes, with plans for surgical intervention when feasible.

## 5 Discussion

Schwannomatosis is a rare, painful clinical syndrome characterized by multiple Schwannomas without NF1 or NF2 features, as seen in this 44‐year‐old patient with masses in the right axilla, bilateral inguinal regions, and right lumbar area. The patient's age aligns with the typical third‐to‐fifth‐decade presentation of Schwannomas [7]. The absence of vestibulocochlear nerve deficits, skin lesions, or gait disturbances distinguishes this case from NF1 and NF2, consistent with literature defining schwannomatosis as a distinct entity [1, 8].

CT imaging prior to chemotherapy revealed well‐encapsulated, heterogeneously enhancing ovoid masses, some with spinal or neuroforamen connections (e.g., “dumb‐bell” shapes in the mediastinal and retroperitoneal masses; Figures 1 and 3), ranging from 4 to 9 cm, aligning with documented schwannoma characteristics [12]. Specific findings included a 6.8 × 6.1 × 4.2 cm mass in the right axilla and mediastinum (Figure 1), bilateral pre‐sacral pelvic masses (Figure 2), a retroperitoneal mass displacing the psoas muscle (Figure 3), and gluteal and inguinal masses (Figure 4). While MRI is the gold standard for soft tissue characterization, showing T1 iso‐hypointense, T2 hyperintense, and heterogeneously enhancing lesions, financial constraints necessitated reliance on CT. Surgery had been done for spinal masses that had an intra‐abdominal component 5 years prior, but recurrence was clearly demonstrated by the clinical examination and the CT imaging at baseline of oncology review. Brain imaging was not done to exclude intracranial stigmata and vestibular involvement to rule out NF2. The lack of brain imaging to exclude vestibular schwannomas and genetic testing are limitations, as these could further confirm the absence of NF2.

Histopathology from different chronological times, multiple sites and laboratories confirmed Schwannomas, supporting the schwannomatosis diagnosis. The patient's chemotherapy response contrasts with literature advocating microsurgical resection, which offers a 95% cure rate with low recurrence [7, 12]. Chemotherapy may reflect resource constraints, as surgery or radiotherapy is preferred for recurrence or inoperable cases [5]. This case highlights the diagnostic utility of CT in low‐resource settings in conjunction with pathology and the need for a high index of suspicion to differentiate schwannomatosis from other PNSTs for optimal management.

## Author Contributions

**Bernadette Pedun:** conceptualization, data curation, formal analysis, investigation, methodology, project administration, resources, software, validation, writing – original draft, writing – review and editing. **Ivaan Pitua:** formal analysis, methodology, visualization, writing – original draft, writing – review and editing. **Felix Bongomin:** formal analysis, methodology, validation, writing – original draft, writing – review and editing. **Samuel Bugeza:** investigation, methodology, project administration, supervision, writing – original draft, writing – review and editing. **Alex Bakenga:** investigation, methodology, validation, writing – original draft, writing – review and editing. **Valeria Nabbosa:** investigation, methodology, supervision, validation, writing – original draft, writing – review and editing.

## Funding

The authors have nothing to report.

## Ethics Statement

The authors have nothing to report.

## Consent

Written informed consent for publication of this case report and accompanying images was obtained from the patient.

## Conflicts of Interest

The authors declare no conflicts of interest.

## Data Availability

All relevant data and materials are included within the text.
